# Evaluating the diagnostic and prognostic value of circulating cathepsin S in gastric cancer

**DOI:** 10.18632/oncotarget.8582

**Published:** 2016-04-05

**Authors:** Wan-Li Liu, Dan Liu, Kai Cheng, Yi-Jun Liu, Shan Xing, Pei-dong Chi, Xiao-Hua Liu, Ning Xue, Yan-zhen Lai, Ling Guo, Ge Zhang

**Affiliations:** ^1^ State Key Laboratory of Oncology in Southern China, Sun Yat-sen University Cancer Center, Guangzhou, China; ^2^ Department of Clinical Laboratory Medicine, Sun Yat-sen University Cancer Center, Guangzhou, China; ^3^ Department of Microbial and Biochemical Pharmacy, School of Pharmaceutical Sciences, Sun Yat-sen University, Guangzhou, China; ^4^ Department of Nasopharyngeal Carcinoma, Sun Yat-sen University of Cancer Center, Guangzhou, China

**Keywords:** Cat S, gastric cancer, diagnosis and prognostic biomarker, ELISA

## Abstract

To evaluate whether serum Cathepsin S (Cat S) could serve as a biomarker for the diagnosis and prognosis of gastric cancer (GC), Enzyme-linked immuno sorbent assay (ELISA) was used to detect serum Cat S in 496 participants including healthy controls and patients with benign gastric diseases, gastric cancer, esophageal cancer, liver cancer, colorectal cancer, nasopharyngeal cancer and lung cancer. The levels of serum Cat S were significantly increased in cancer patients, especially in GC patients. The qRT-PCR, Western blotting, and immunohistochemical staining revealed the overexpression of Cat S in GC cell lines and tissues. The diagnostic value of serum Cat S for GC patients from controls resulted in an AUC of 0.803 with a sensitivity of 60.7% and a specificity of 90.0%. Moreover, the levels of serum Cat S were associated with GC tumor volume, lymphoid nodal status, metastasis status, and stages. Moreover, the patients with high levels of serum Cat S had a poorer overall survival. Univariate analysis revealed Cat S expression was a prognostic factor. The knockdown of Cat S significantly suppressed the migration and invasion of GC cells. This study suggested serum Cat S may be a potential biomarker for the diagnosis and prognosis of GC.

## INTRODUCTION

Gastric cancer (GC) remains the second-most frequent cause of cancer-related death worldwide and is particularly prevalent in Asia [1]. Currently, invasive tests, such as endoscopy and biopsy, are widely used as the diagnosis criteria for gastric cancer, but such tests are limited due to their high cost and suffering. Serum biomarkers, for example, CEA, CA72-4, CA19–9, lack sufficient sensitivity and specificity. Therefore, there is a growing need to identify useful biomarkers for early non-invasive diagnosis and monitoring of the progression of gastric cancer.

Our understanding of cysteine cathepsin proteases originates with their canonical role as degradative enzymes of the lysosome. They are now recognized to play pivotal roles in cancer progression, such as invasion, metastasis and angiogenesis [2, 3]. Cathepsin S (Cat S) is unique amongst the cysteine cathepsin family due to restricted expression associated with antigen presenting cell (APCs) [4]. Cat S is a secreted protein and it has two forms, including precursor and mature form. Biochemically, it retains the catalytic activity over a broad pH range (4.5-8.0) [5], thus Cat S has more physiological importance than other cysteine cathepsins in the processing of proteins in the extracellular microenvironment [6]. Previous studies have found deregulated expression of Cat S in a range of human tumor types including lung, brain, colorectal and prostate carcinomas [7–14]. Cat S has been shown to possess an important role in cancer progression, such as angiogenesis [15], invasion and volume [3, 9], and these processes are enabled by the ability of Cat S to degrade the extracellular matrix, affect inflammation and the immune response [16, 17], and regulate other tumourigenic factors [18]. Moreover, the expression of Cat S by TAMs is critical for promoting tumor growth and metastasis [19]. Indeed, Cat S demonstrated to have more cancer-related functions than the other family members.

The cathepsin family plays an important role in the progression of gastric cancer. High serum levels of Cat B in gastric cancer are associated with advanced tumor stages and progressive diseases [20]. Additionally, *Helicobacter pylori* (*H. pylori*) infection has been investigated in the context of up-regulated Cat X in gastritis and gastric cancer [21]. Recently, patients with gastric cancer have been found to exhibit significantly increased Cat D expression [22]. High Cat L expression has been found in gastrointestinal stromal tumors [23]. Cat E can serve as a marker of gastric differentiation and signet-ring cell carcinomas of the stomach [24]. Moreover, Cat S has been found to be overexpressed in gastric cancer tissues and to mediate gastric cancer cell migration and invasion though a putative network of metastasis-associated proteins [25]. However, the clinical significance of serum Cat S in the diagnosis and progression of gastric cancer remains unclear.

In the present study, we measured the expression and the levels of Cat S in GC cell lines, GC tissues and serum from GC patients. We investigated the associations of the levels of serum Cat S with the patients’ clinical outcomes to assess whether serum Cat S can work as a valuable diagnostic and prognostic biomarker for GC.

## RESULTS

### Higher levels of Cat S were detected in the serum of cancer patients, particularly the gastric cancer patients

**Figure 1:**
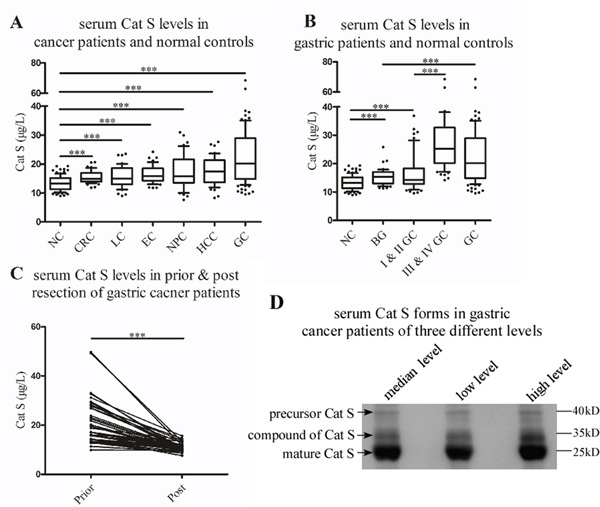
Higher levels of Cat S were detected in the serum of cancer patients, particularly the gastric cancer patients **A.** Significant difference (*P* < 0.001) was found in the pairwise comparison between normal controls and cancer patients. **B.** Distinct comparation (*P* < 0.001) of Cat S was found between healthy controls and benign gastric diseases. Cat S levels of early stages (I & II) GC patients were lower than those of late stage (III & IV) GC patients, but higher than normal controls (*P* < 0.001). GC patients had higher serum Cat S levels than those of benign gastric disease patients (*P* < 0.001). **C.** The levels of Cat S decreased after tumor resection with significant difference (*P* < 0.001). **D.** Western blotting analysis of Cat S expression in three different gastric cancer patients’ serum. There were three bands, including the precursor form (37kD), the mature form (24kD) and a compound of Cat S and Cys-C, probably (35kD). “***” represents “*P* < 0.001”. “NC” is short for “normal control”. “CRC” is short for “colorectal cancer”. “LC” is short for “lung cancer”. “EC” is short for “esophagus cancer”. “NPC” is short for “nasopharyngeal carclnoma”. “HCC” is short for “hepatocellular carcinoma”. “GC” is short for “gastric cancer”. “BG” is short for “benign gastric diseases”.

We detected the levels of total serum Cat S in the healthy controls (n = 99) and the patients with a variety of cancers (n = 347) that included gastric cancer (n = 119), esophageal cancer (n = 45), liver cancer (n = 46), colorectal cancer (n = 48), nasopharyngeal cancer (n = 47), and lung cancer (n = 42) by ELISA. The serum Cat S values from the cancer patients were significantly higher than those from the healthy controls (*P* < 0.001; Figure 1A), and the highest mean serum Cat S value was observed in the patient group with gastric cancer.

Furthermore, we explored the levels of total serum Cat S in patients with benign gastric diseases (n = 49). Interestingly, these levels were higher than those of the normal controls but lower than those of the GC patients. The levels of Cat S in early-stage GC patients (*i.e.*, stages I and II) were lower than those in late-stage GC patients (*i.e.*, stages III and IV) but greater than those in the controls. All of these differences were significant (*P* < 0.001; Figure 1B).

To assess the levels of serum Cat S before and after tumor resection, we collected serum both pre- and post-operation. As demonstrated in Figure 1C, the levels of Cat S significantly decreased after the operations (*P* < 0.001). All of the results are listed in Table 1.

**Table 1:** The mean value and standard deviation of Cat S in different population

Group	Cases	Age median (range)	Gender (M/F)	Cat S (μg/L) Mean ±Sd.
Healthy controls	99	53(23-79)	31/68	13.44±2.46
Benign gastric diseases	49	55(39-77)	33/16	15.36±2.77
Gastric cancer (GC)	119	56(24-81)	86/33	22.83±10.48
I & II stage GC	53	57(25-81)	38/15	16.67±6.19
III & IV stage GC	66	55(24-81)	48/18	27.77±10.64
Pre-treatment GC	42	52(25-79)	30/12	20.85±9.18
Post-treatment GC	42	52(25-79)	30/12	10.89±1.71
Colorectal cancer	48	55(39-76)	32/16	15.53±2.13
Lung cancer	42	54(33-80)	31/11	15.63±3.69
Esophageal cancer patients	45	52(29-76)	32/13	16.41±2.96
Nasopharyngeal cancer	47	56(30-75)	35/12	17.32±5.66
Liver cancer	46	54(31-74)	33/13	17.63±4.63

The precursor and mature form of serum Cat S were detected by Western blotting with an antibody that can recognize the two forms. We found the major form of Cat S was the mature one, and in contrast, the bands of the precursor were very weak. The brightness of the bands was in consistent to the preceding ELISA values and the two forms were in the same trend. Moreover, there was an obvious extra bands about 35-kD beyond the mature form. We hypothesized that it was the compound of Cat S and other matter in serum, probably cystatin C, the most abundant extracellular inhibitor of cysteine proteases with molecular weight about 13-kD (Figure 1D).

### Diagnostic value of serum Cat S in GC patients

**Figure 2:**
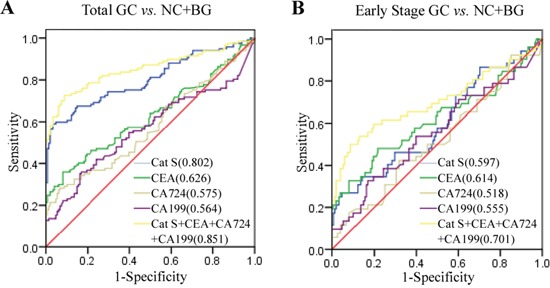
Diagnostic value of serum Cat S in GC patients Figures in the brackets were the AUCs from statistical analyses by SPSS software. **A.** The ROC curves demonstrated the diagnostic strength of Cat S, CEA, CA724, CA199 and a combination of four of them in identifying GC from controls (healthy controls and benign gastric diseases). **B.** The ROC curves of serum Cat S, CEA, CA724, CA199 and a combination of four of them in diagnosis of early stage GC against former controls.

The serum Cat S level was able to differentiate GC patients (n = 119) from healthy controls (n = 99) and the patients with benign gastric diseases (n = 49) with an AUC of 0.802 based on a ROC analysis. In contrast, the AUCs of the traditional biomarkers of CEA, CA724 and CA199 were 0.626, 0.575, and 0.564, respectively. The sensitivity of Cat S was as high as 60.7%, and the specificity reached 90.0%. The specificities of the traditional biomarkers were also very high, but their sensitivities were not sufficient. The combination of Cat S, CEA, CA724 and CA199 resulted in a better AUC of 0.851 with a specificity of 91.2% and a sensitivity of 72.6% (Figure 2A). The statistical analysis revealed that the effectiveness of Cat S in the diagnosis of GC was better than those of the traditional markers of CEA, CA724 and CA199.

The differentiation of early-stage GC patients from benign gastric disease patients with high sensitivity and specificity has always been a critical problem. In our study, we also conducted a ROC analysis to access the ability of Cat S to distinguish between 53 stage I and II GC patients and 148 controls. As shown in Figure 2B, the AUCs for Cat S, CEA, CA724 and CA199 were not sufficient, but the AUC for the combination of these factors reached 0.701 with a specificity of 91.2% and a sensitivity of 50.0%. The details are presented in Table 2.

**Table 2:** Diagnostic values of the Cat S, CEA, CA724, CA199 and their combination

		Cut-off value	AUC	*P*	Sensitivity (%)	Specificity (%)
GC *vs.* NC+BG	Cat S	17.38	0.802	<0.001	60.7	90.0
	CEA	5.00	0.626	<0.001	24.8	98.0
	CA724	5.3	0.575	0.035	21.4	95.9
	CA199	35	0.564	0.072	12.7	99.3
	Cat S + CEA + CA724 + CA199	0.44	0.851	<0.001	72.6	91.2
Early GC *vs.* NC+BG	Cat S	18.07	0.597	0.037	26.9	95.3
	CEA	5.00	0.614	0.015	21.2	98.0
	CA724	5.3	0.518	0.695	7.7	97.0
	CA199	35	0.555	0.237	9.6	99.4
	Cat S + CEA + CA724 + CA199	0.34	0.701	<0.001	50.0	91.2

“NC” is short for “normal control”. “BG” is short for “benign gastric diseases”.

### Association between serum Cat S levels and the progression of GC

The associations between the serum Cat S levels and the clinicopathological parameters are presented in Table 3. The Cat S levels were not associated with clinicopathological parameters including age, gender, smoking status, alcohol intake or histological differentiation, but significant correlations were found with T classification, lymph node metastasis, distant metastasis and clinical stage (*P* < 0.001). Our findings suggested that increased levels of serum Cat S were associated with GC development and progression.

**Table 3:** Clinical characteristics of the gastric cancer patients

Characteristics	No. of patients	Levels of Serum Cat S		*P*
		low	high	
patients	119	43	76	
Age				
Median	56			
Range	24-81			
≤ 56	60	18	42	
> 56	59	25	34	0.16
Gender				
Female	33	13	20	
Male	86	30	56	0.64
Smoking Status				
Smoker	38	11	27	
Non-smoker	81	32	49	0.26
Alcohol Intake				
Yes	23	5	18	
No	96	38	58	0.11
Grade				
Grade 1	4	3	1	
Grade 2	31	11	20	
Grade 3	84	29	55	0.257
pT Status				
pT 1	15	14	1	
pT 2	20	13	7	
pT 3	40	13	27	
pT 4	42	3	39	<0.001
pN Status				
pN 0	39	30	9	
pN 1/2/3/X	80	13	67	<0.001
pM Status				
pM 0	100	43	57	
pM 1/X	19	0	19	<0.001
Stage				
I–II	53	37	16	
III-IV	66	6	60	<0.001

*P* values were calculated by Pearson's Chi-square test.

Moreover, some researchers have demonstrated the involvement of Cat S in atherosclerosis [26] and dyslipidemia [27]. The associations of the median serum Cat S levels with the serum lipid profile, inflammatory biomarker CRP and the renal function biomarker Cys-C are presented in Table 4. The serum Cat S levels were not correlated with TG, HDL-C, LDL-C, GLU, apoA1 or apoB. The inflammatory biomarker CRP and the renal function biomarker Cys-C were also unrelated to Cat S. These results indicated that angiocardiopathy, dyslipidemia, inflammation and renal diseases almost had little effect on the serum Cat S levels in the GC patients.

**Table 4:** Expression of Cat S with different characteristics of biochemical parameters

Clinical parameters	No. of patients	Levels of Serum Cat S		*P*
		low	high	
CRP (mg/L)				
≤ 5	58	22	36	
> 5	53	18	35	0.664
Cys-C (mg/L)				
≤ 1.5	111	40	71	
> 1.5	0	0	0	…
≤ 1.09	93	33	60	
> 1.09	18	7	11	0.783
GLU (mmol/L)				
≤ 6.01	77	29	48	
> 6.01	34	11	23	0.591
TG (mmol/L)				
≤ 1.7	91	34	58	
> 1.7	20	6	13	0.657
HDL-C (mmol/L)				
≤ 2.2	110	39	71	
> 2.2	1	1	0	0.181
LDL-C (mmol/L)				
≤ 3.4	83	28	55	
> 3.4	28	12	16	0.385
apoA1 (g/L)				
≤ 1.76	111	40	71	
> 1.76	0	0	0	…
≤ 1.45	105	38	67	
> 1.45	6	2	4	0.887
apoB (g/L)				
≤ 1.14	98	34	64	
> 1.14	13	6	7	0.419

*P* values were calculated by Pearson's Chi-square test. “…” represent “data not available”. The clinical cut-off value of Cys-C and apoA1 could not divide the patients with an available *P* value, so the The cut-off values were defined as the value with the maximization of the Yuden index.

### Correlation of the serum Cat S levels and overall survival

**Figure 3:**
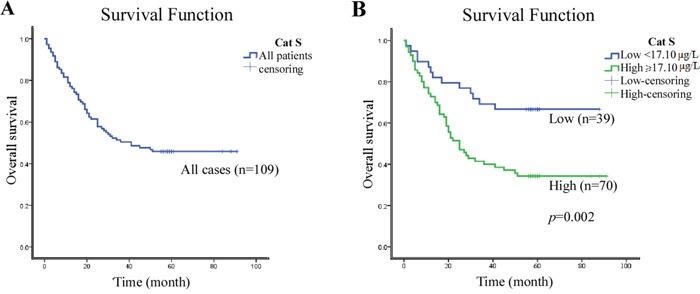
Kaplan–Meier estimates of the probability of survival **A.** The five-year overall survival (OS) rate was 44.0% of 109 GC patient; **B.** High Cat S expression level was significantly correlated to poor OS (*P* = 0.002) in all GC patients.

Among the 119 GC patients in this study, the median follow-up period was 37.3 months (range, 1 to 91 months), and 59 cancer-related deaths were recorded by the final clinical follow-up. The 5-year overall survival (OS) rate was 44.0% for the total study population (Figure 3A). Kaplan–Meier analysis revealed that the OS was longer for the patients with low serum Cat S levels than those with high Cat S levels (*P* = 0.002, median 60.7 *vs.* 17.4 months, Figure 3B).

Next, we examined the OS using a Cox proportional hazards model to determine the independent predictors. A series of factors, including age, gender, smoking status, alcohol intake, grade, pathological staging and Cat S expression, were entered into the univariate Cox regression analyses. The variables that were found to be significant in the univariate analyses were further examined in the multivariate analysis. The univariate analyses revealed that the TNM stage and Cat S expression were prognostic factors for overall survival, but the multivariate analysis model revealed that only the TNM stage was an independent predictor of the OS (Table 5).

**Table 5:** Univariate analysis and multivariate analysis for predictors of overall survival

Variables	Univariate analysis			Multivariate analysis		
	HR	CI	*P*	HR	CI	*P*
Age	1.268	0.761-2.115	0.362	…	…	…
Gender	0.912	0.513-1.691	0.752	…	…	…
Smoke	1.525	0.899-2.588	0.117	…	…	…
Alcohol Intake	0.988	0.513-1.903	0.972	…	…	…
Grade	1.029	0.625-1.694	0.91	…	…	…
TNM stage	3.692	1.984-6.871	<0.001	4.865	2.299-10.294	<0.001
Cat S	2.555	1.377-4.739	0.003	1.333	0.606-2.932	0.474

Variables: age, >56 *vs.* ≤56; Gender, female *vs.* male; smoke, smoker *vs.* non-smoker; alcohol consumption, yes *vs.* no; grade, grade 3 *vs.* grade 2 *vs.* grade 1; stage, III & IV *vs.* II & I. “…” represent “data not available”. “HR” is short for “hazard ratio”. “CI” is short for “confidence interval”.

### Cat S expression was up-regulated in GC cell lines and GC tissues

**Figure 4:**
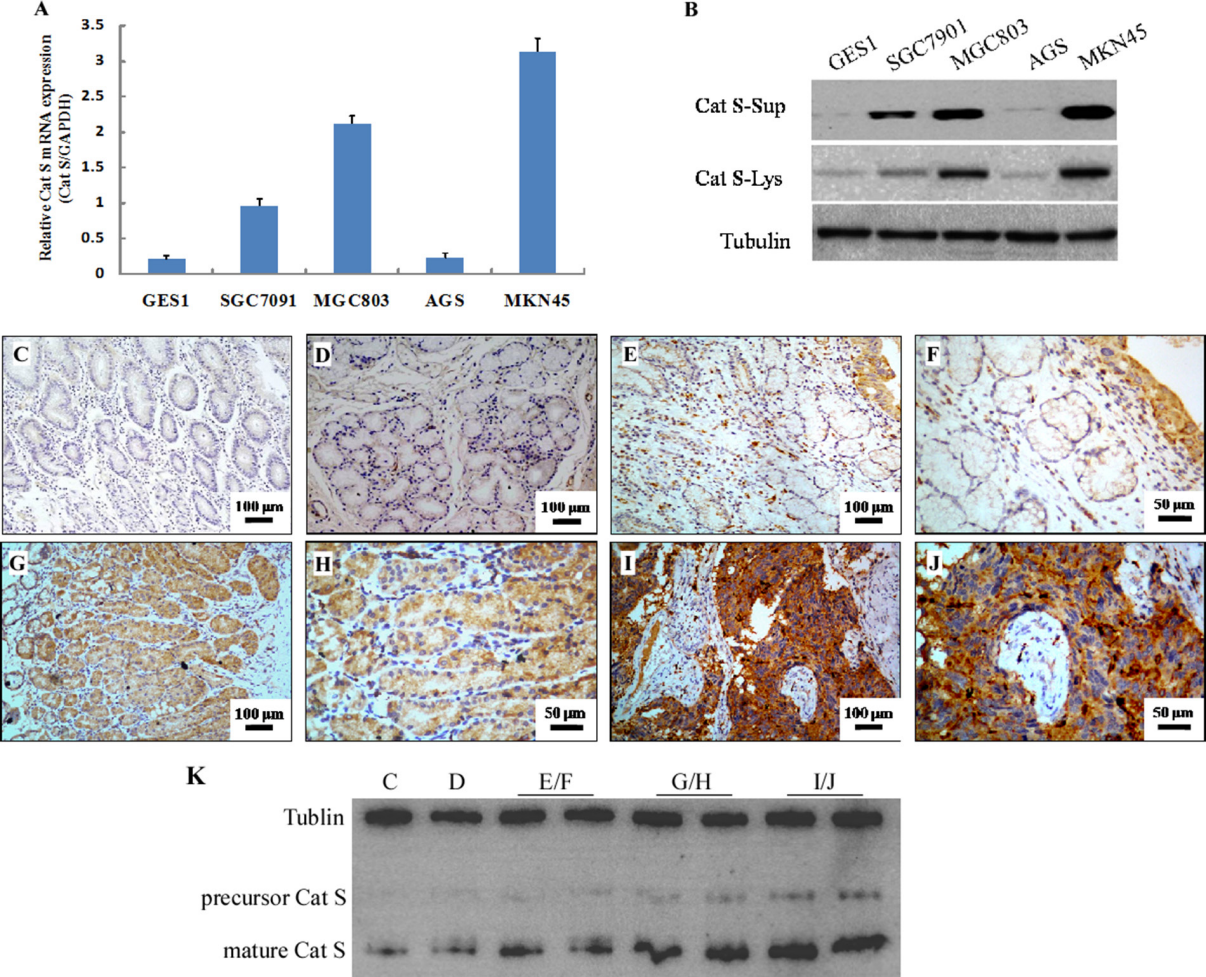
Cat S expression was up-regulated in GC cell lines and GC tissues Cat S mRNA relative expression **A.** and protein levels from cell lysate and supernatant **B.** were compared between a panel of GC cell lines including SGC7091, MGC803, AGS and MKN45, and the immortalized normal human esophageal epithelial cell line, GES1. The mRNA levels are presented as means ± Sd. and normalized to the housekeeping gene GAPDH in qRT-PCR. The internal control was Tubulin in Western blotting. Representative Cat S immunohistochemistry staining in gastric tissues 100× **C, D, E, G, I.** and 200× **F, H, J. C, D, E, F.** Negative or weak positive expression of Cat S staining in the normal stomach. **G, H.** Intestinal type gastric cancer cells over expressed Cat S throughout the cytoplasm. **I, J.** Diffuse type gastric adenocarcinoma sections had cytoplasm positive staining of Cat S. **K.** Western blotting analysis of Cat S expression in tumor and normal stomach. The samples were in line with the immunohistochemistry tissue. There were only two bands, namely the precursor form (37kD) and the mature form (24kD). The internal control was Tubulin.

To investigate the Cat S expression in the GC cell lines, qRT-PCR and Western blotting were performed in both an immortalized normal human gastric epithelial cell line, *i.e.*, GES1, and a panel of gastric cancer cell lines that included GES1, SGC7091, MGC803, AGS and MKN45. Cat S mRNA and protein were barely detected in GES1, while significantly elevated expressions were detected in all of the GC cell lines with an exception of the AGS line, which was a gastric adenocarcinoma cell line (Figure 4A and 4B). Notably, higher levels of secreted Cat S were observed in the supernatant of the gastric cancer cell lines, which were in accordance with the cell lysate expressions. The mature Cat S was the major form in the cell lysate and supernatant. In conclusion, Cat S was frequently up-regulated in the GC cell lines.

To evaluate Cat S expression *in vivo*, we conducted immunohistochemistry with an antibody against human Cat S using prepared paraffin sections from pathological archives. Absent or weak Cat S staining was detected in the normal tissues as illustrated in representative images in Figure 4C, 4D, 4E and 4F. In contrast, Cat S protein was detected in 17 of the 20 GC samples (85.0%). Cat S immunoreactivity was observed at various levels, and the major localization was observed in the cytoplasm of the tumor cells in both intestinal (Figure 4G and 4H) and diffuse type adenocarcinoma sections (Figure 4I and 4J). Additionally, Cat S staining was also found in some cells within the tumor stroma; these Cat S-positive cells may have been APCs, such as macrophages and dendritic cells, as described in a previous study [28].

Besides, Western blotting was again applied to normal and GC tissues to detect the expression of Cat S. We found the main form was still the mature one and the brightness of the bands were in consistent to the immunohistochemistry results (Figure 4K).

### Knockdown of Cat S suppressed the migration and invasion of GC cells

**Figure 5:**
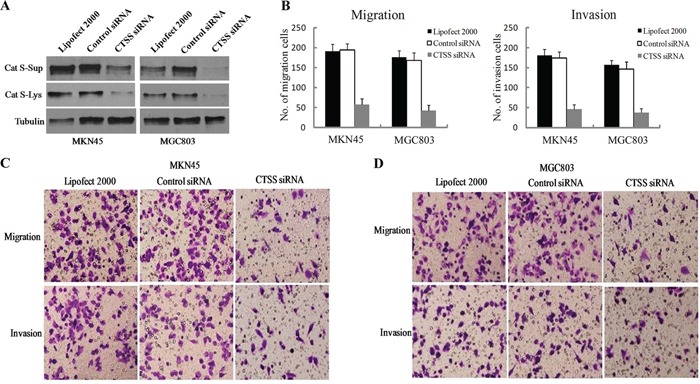
Knockdown of Cat S suppressed the migration and invasion of GC cells **A.** Western blotting of cell lysate and supernatant from MKN45 and MGC803 cells transfected with NC or Cat S-targeting siRNAs, with Tublin as an internal control showed the effectiveness of Cat S knockdown by siRNA. **B.** Statistical graph of adherent cells presented as mean number±Sd. by triplet results of transwell migration and matrigel invasion assays. Representative staining images (× 100) of adherent cells in transwell migration assays and transwell matrigel invasion assays in MKN45 **C.** and MGC803 **D.** GC cell lines.

Previous study has found that Cat S can mediate gastric cancer cell migration and invasion in MKN7 cell line [25]. Again to approve the roles of Cat S in the migration and invasion of GC cells, the establishment of the Cat S-depletion in MKN45 and MGC803 was undertaken using siRNA constructs specific for Cat S in parallel with a control. The downregulation of Cat S was confirmed with a Western blotting assay in both cell lysate and supernatant, which indicated that the Cat S-specific siRNA construction yielded an efficient knockdown of Cat S protein levels (Figure 5A). To functionally characterize the roles of Cat S, the cell migration and invasiveness abilities were assessed using transwell assays. The knockdown of Cat S clearly reduced both the migration ability and the invasive nature of the GC cells and even elicited significant reductions compared to the wild-type Cat S cells (Figure 5C and 5D). The statistical analyses were conducted on triplet results. The significant differences are shown in Figure 5B.

## DISCUSSION

In the current study, we aimed to bridge the gap between Cat S and clinical applications by evaluating circulating Cat S levels and comparing those results to clinic information. The serum Cat S levels were found to be significantly elevated in the cancer patients, including those with gastric cancer, esophageal cancer, liver cancer, nasopharyngeal cancer, lung cancer and colorectal cancer. Among these patients, the patients with GC exhibited the highest mean level of serum Cat S. Many of the members of the cathepsin family have been investigated as tumor markers, so we further explored the clinical significance of serum Cat S in gastric cancer.

A panel of gastric cancer cell lines revealed high levels of Cat S expression both in terms of mRNA and protein. Moreover, elevated secreted Cat S was also found in the cell supernatant of most of the gastric cancer cell lines compared with immortalized gastric cell line. The results of our study are consistent with those of a previous study that found that Cat S is overexpressed in the supernatant of GC cell lines as identified by proteomics [25].

Cat S is expressed by APCs, including macrophages, B-lymphocytes, dendritic cells and microglia [29, 30]. Cat S immunohistochemical staining has been used as a marker of lesion macrophages [30]. Cat S has also been determined to be overexpressed in the tumor cells of tissues including hepatocellular carcinoma [31], lung cancer [32], and colorectal cancer [33]. However, in other tumor types, such as prostate tumor [34] and sinonasal inverted papilloma [35], Cat S is primarily expressed by tumor-infiltrating macrophages but not tumor cells. In our study, we revealed Cat S was strongly expressed in GC tissues, and the major form was the mature one. Cat S was primarily located in the cytoplasm of the tumor cells. In the normal nonmalignant tissue adjacent to the cancer, weak or the absence of Cat S staining was detected. All of our studies led to the conclusion that gastric tumor cells overexpress Cat S and thus contributed to the extremely high levels of circulating Cat S in patients with gastric cancer. Additionally, the significant decrease in serum Cat S levels after tumor resection also proved that serum Cat S may be a valuable tumor biomarker for GC.

Cat S is a secreted protein, and thus, distinct serum levels make it a potential diagnostic marker of some diseases [36, 37]. In this study, we revealed that the effectiveness of Cat S in the differentiation of GC patients from those with benign gastric diseases and normal controls was superior to the traditional markers of CEA, CA724 and CA199, although the sensitivity of the serum Cat S was not particularly high. However, the combination of Cat S and these three biomarkers resulted in great improvement in the sensitivity of the GC diagnosis. Furthermore, the combination of the four markers enabled more effective differentiation of early-stage GC patients from those with benign gastric diseases and the normal controls compared with the use of any single marker. Currently, new non-invasive blood tests are urgently needed for the early diagnosis of gastric cancers. Our study suggested that serum Cat S could be a potential biomarker for the diagnosis of GC and even early-stage GC.

Serum Cat S levels have been shown to be increased in diabetes [38], atherosclerosis [36], myocardial infarction [39], and adiposity [40], when compared with normal healthy controls. Moreover, the strong correlation between serum Cat S levels and high-sensitivity C-reactive protein (hs-CRP) or Cys-C was proved in many diseases like abdominal aortic aneurysm patients [41, 42]. In our study, there were no significant associations between the median serum Cat S level and the serum lipid profile, *i.e.,* TG, HDL-C, LDL-C, GLU, apoA1 and apoB. Serum Cat S was not correlated with the inflammatory biomarker CRP or with the renal function biomarker Cys-C. It is known that the most abundant extracellular inhibitor of cysteine proteases is Cys-C. In our study, we found an obvious extra band beyond the mature band. The molecule weight seemed the compound of mature Cat S and Cys-C, but we did not detect it further. Cox *et al.* have described a robust and powerful method to detect the level and activity of mature Cat S in human serum. They observed that only 0.4 to 1.1% of circulating Cat S was enzymatically active, which was caused by the binding to its endogenous inhibitor cystatin C [43]. But in this study we did not detect the activity of Cat S in gastric cancer patients’ serum and thus did not know how much the activity of Cat S was inhibited by cystatin C. These results indicated that angiocardiopathy, dyslipidemia, inflammation and renal diseases had almost little effect on serum Cat S in GC. Thus Cat S achieves well diagnostic capacity for little interference. *H. pylori* infection has been found to up-regulate Cat X in gastritis and gastric cancer [21]. However, in our study, we did not examine the association between Cat S and *H. pylori* infection, which would require further investigation.

Additionally, we found associations of the serum Cat S levels with the GC patients’ tumor sizes, lymph node stages and distant metastases; *i.e.,* more advanced TNM stages were associated with higher levels of serum Cat S. To further define the clinical importance of Cat S, we investigated the correlation between Cat S expression and the overall survival of the GC patients. The patients with high levels of serum Cat S exhibited an inferior overall survival. These results are in accordance with those of reports that have demonstrated oncogenic Cat S in a variety of cancers, such as astrocytomas [11] and colorectal cancer [13], and the patients with higher Cat S expression levels were also found to exhibit worse survival. The univariate analyses revealed that Cat S expression was a prognostic factor along with the TNM stage. However, Cat S was not found to be an independent predictor of OS in the multivariate analysis model, which was probably due to its close correlation with the TNM stage.

During tumor development and progression, one of the most important events is local invasion, which is mediated by the degradation of ECM components, and the proteases involved in this process, along with their inhibitors, are increasingly being identified. Different types of malignant human neoplasms, such as colon carcinomas [44], breast [45, 46], lung [47], larynx tumors [48], melanomas [49] and other types of malignancies, can produce cathepsins that degrade the ECM and thus increase the cancer cell invasion [50]. Recently, multiple studies have indicated that Cat S not only plays a crucial role in antigen presentation but also acts as an important protagonist in the invasion of cancers [15]. In our study, Cat S was found to play an important role in gastric cancer cell migration and invasion. These findings are consistent with those of other reports that have demonstrated Cat S cleaves extracellular adhesion proteins to drive cell migration and invasion and that Cat S promotes gastric cancer cell migration and putatively mediates invasion via a network of metastasis-associated proteins [25]. The functions of Cat S in tumor cell migration and invasion may contribute to the role of Cat S as an indicator of worse prognoses in GC.

In summary, we observed high levels of expression of Cat S in GC, and the knockdown Cat S suppressed GC cell migration and invasion. These results demonstrated that serum Cat S could be a useful biomarker in the diagnosis and prognosis of GC. Because Cat S is a member of a large group of extracellular proteases that are attractive drug targets, Cat S may be a potential therapeutic target for patients with GC.

## MATERIALS AND METHODS

### Patients, serum and tissues

The serum were collected between January 2010 and January 2011 from 347 patients, including gastric cancer (n = 119), esophageal cancer (n = 45), liver cancer (n = 46), colorectal cancer (n = 48), nasopharyngeal cancer (n = 47), and lung cancer patients (n = 42) at the time of diagnosis before tumor resection at the Cancer Center of Sun Yat-sen University (SYSUCC). Serum samples from 42 cases were collected pre-operatively and at 2 weeks post-operation. All cancers were diagnosed based upon the histopathology examination. The pathological stages of the gastric cancers were recorded according to the Union for International Cancer Control/American Joint Committee on Cancer (UICC/AJCC) classification (2010). Follow-up information was available for 109 of the patients with gastric cancer.

The serum of 49 benign gastric disease patients, including 11 cases of chronic gastritis, 19 cases of gastric ulcer, 11 cases of chronic atrophic gastritis and 8 cases of polyps of the stomach, were collected before treatment at the First Affiliated Hospital of Sun Yat-sen University. The age range of the patients was from 39 to 77, and the median age was 55. There were 33 males and 16 females. The serum from 99 healthy volunteers (31 males and 68 females) ranging in age from 23 to 79 years (mean: 53 years) were collected. The serum of healthy donors was collected at the physical examination department of the Cancer Center of Sun Yat-Sen University. The control group was matched as closely as possible to the gastric cancer group in terms of sex, age, previous handling and the time period of sample collection.

A 5-ml blood sample from each participating patient and health control was allowed to clot for 30 to 60 min at room temperature. Each clotted sample was centrifuged at 2,500 g for 10 min. All sera were aliquoted and frozen at -70°C until use.

For the immunohistochemistry and Western blotting analyses, GC tissues and matched carcinoma-adjacent tissues were collected and divided to lysate and paraffin-embed from SYSUCC.

Prior to the use of these clinical materials for the investigation, informed consent from the patients and approval from the institute's Research Ethics Committee were obtained.

### ELISA assay of serum Cat S

Serum Cat S levels were determined using a sandwich enzyme immunoassay (ELISA) (human cathepsin S-total, DY1183; R&D Systems, Minneapolis, Minnesota) according to the manufacturer's instructions. In brief, serum samples were diluted 1:10 with 0.9% BSA (Proliant, Lot: BB70480101), and then added to the pre-coated ELISA plate (100 μl per well). Plates were incubated overnight at 4°C, washed and then incubated with conjugated solution for 2h at 37°C. Plates were then developed by treatment with 3,3′,5,5′-tetramethylbenzidine (TMB) and H_2_SO_4_ solution according to the manufacturer's instructions of the ELISA kit. The serum concentrations of Cat S were determined from standard curves derived from the provided calibrators, and ranged from 100 to 1000 pg/ml.

### Tumor markers and biochemical test items

The concentrations of CEA, CA724 and CA199 in the serum were assessed using electrochemiluminescence immunoassay (ECLIA) kits (CEA, lot: 172356; CA724, lot: 169393; CA199, Roche, German) on a Roche E170 fully automatic electrochemistry luminescence immunity analyzer (Roche, German). Each test included a standard control (CV < 5%).

The serum biochemical indices including TG, HDL-C, LDL-C, GLU, apoA1, apoB, inflammatory biomarker CRP and renal function biomarker Cys-C were measured using accorded reagent kits (WAKO, Japan) on an automatic biochemical analyzer (LABOSPECT_008; Hitachi High Technologies Corporation, Tokyo, Japan).

### Western blotting analysis

Total protein was extracted by using a lysis buffer and protease inhibitor (Beyotime Biotechnology, China). Equivalent protein amounts were denatured in an SDS sample buffer, and then were separated by SDS-PAGE and transferred onto polyvinylidene difluoride membrane. After being blocked with 5% non-fat dry milk in PBS containing 0.05% Tween-20, the blotted membranes were incubated with anti-human Cat S antibody (sc74429, Santa Cruz; 1:100) and then secondary antibody (1:5000, Boster, China). α-tublin protein levels also were determined by using the specific antibody (1:3000, Abcam, UK) as a loading control.

### Immunohistochemistry

The paraffin-embedded GC tissues were sectioned into 4-μm-thick sections. The sections were dewaxed, rehydrated and rinsed. The antigens were retrieved by heating the tissue sections at 100°C for 20 min in citrate (10 mmol/L, pH 6.0) solution when necessary. The sections were then immersed in a 3% hydrogen peroxide solution for 10 min to block endogenous peroxidase activity and incubated with the primary antibody goat anti-human Cat S antibody (AF1183, R&D Systems; 1:50) at 4°C overnight. A negative control was performed by replacing the primary antibody with PBS. The sections were then incubated with a horseradish peroxidase labeled secondary antibody (Zymed, Ready-to-use) at room temperature for 120 min. Finally, the signal was developed for visualization with 3, 3′-diaminobenzidine tetrahydrochloride, and all of the slides were counterstained with hematoxylin.

### Cell lines

The gastric cancer cell lines, SGC7901, MKN45, AGS, MGC803, and the gastric epithelial mucosa cell line GES1 were obtained from the Committee of Type Culture Collection of Chinese Academy of Sciences (Shanghai, China). The cell lines were cultured in RPMI 1640 media supplied with 10% heat-inactive fetal bovine serum (FBS). The cells were incubated at 37°C in a humidified chamber containing 5% CO_2_.

### RNA extraction and real-time quantitative PCR

Total RNA was extracted from cell lines using the Trizol reagent (Invitrogen, USA) according to the manufacture's instruction. Reverse transcription of total RNA (1 μg) was done using SuperScript II reverse transcriptase. The quantification of target and reference gene (GAPDH) were performed in triplicate on a LightCycler^®^ 480 II (Roche, Applied Science) using a SYBR green-based assay (BioRad, USA). Expression data were normalized to the geometric mean by housekeeping gene GAPDH as an internal control. The primers used in the real-time RT-PCR reaction were as follows: Cat S: 5′-GCCTGATTCTGTGGACTGG-3′ (forward), 5′-GATGTACTGGAAAGCCGTTGT-3′ (reverse); and GAPDH, 5ʹ-GACTCATGACCACAGTCCATGC-3ʹ (forward) and 5ʹ-AGAGGCAGGGATGATGTTCTG-3ʹ (reverse).

### The siRNA transfection, cell migration and invasion assays

MKN45 and MGC803 cells were first trypsinized in serum free media and counted before transfected with control siRNA (Invitrogen, China) or Cat S targeted siRNA using RNAiMAX (Invitrogen), according to the manufacturer's instructions. The Cat S siRNA sequence used was 5ʹ-AGAAUCAGGCAAUAUCCGAUUAGGG-3ʹ. Two days following transfection, cell migration and invasion assays were conducted. Cell migration was analyzed by a transwell chamber assay. Cell invasion assays were performed using BD BioCoat™ Matrigel™Invasion Chambers. FBS (10%) was used as the chemoattractant. Cells on the lower surface of the insert were fixed and stained followed by counting under a light microscope. Cells were visualized using Olympus BX50 microscope (Olympus Opticol Co.), ten representative field of view images were taken using Nikon Digital Sight DS-U2 (Nikon, Japan) and NIS elements F3.0 software was used (Nikon). Experiments were performed at least in triplicates.

### Statistical analysis

All statistical analyses were carried out using the SPSS 16.0 statistical software package (SPSS Inc., Chicago, IL). The Mann-Whitney U test was used to evaluate the difference in serum Cat S concentrations between tumor patients and healthy controls. Pearson's chi-squared test was used to analyze the relationship between Cat S levels and patients’ and clinical parameters’ characteristics. The efficacy of diagnosis for gastric cancer was evaluated by the area under receiver operating characteristic (ROC) curve (AUC). The cut-off values were defined as the value with the maximization of the Yuden index. Furthermore, sensitivity, specificity were used to compare the efficiency of diagnosis. Survival curves were plotted by the Kaplan-Meier method and compared using the log rank test. The significance of various variables for survival was analyzed using the Cox proportional hazards model (univariate and multivariate analysis). All statistical tests were two-sided, *P* <0.05 was considered to be statistically significant in all cases.
